# Leveraging space–time modulation for energy coupling control in electromagnetic coupled transmission lines structures

**DOI:** 10.1038/s41598-023-28925-1

**Published:** 2023-02-02

**Authors:** Mohammad Baharian, Jalil A. Rashed Mohassel

**Affiliations:** Center of Excellence On Applied Electromagnetic Systems, School of ECE, College of Engineering, University of Tehran, Tehran, Iran

**Keywords:** Engineering, Electrical and electronic engineering

## Abstract

This article presents a technique for controlling energy coupling in a coupled transmission line system based on the space–time modulation concept. The per-unit-length mutual capacitance and mutual inductance of the structure are modulated in space and time. The main idea is based on the harmonic generation property of space–time modulated media. As the amplitude of harmonics is a function of modulation parameters it is demonstrated that by choosing an appropriate space–time modulation scheme energy of different harmonics can be engineered leading to crosstalk reduction. In the quest for designing an effective space–time modulation scheme, an analytical method is developed for the examination of the space–time modulated coupled transmission line. The proposed method which is based on the state space formulation and benefits from the coupled mode theory is fast and accurate making it feasible for design problems. To validate the proposed analytical method, a full-wave simulation method has been used. The time-varying nature of the problem makes the finite-difference-time-domain the most appropriate choice. The validity of the analytical method is rigorously verified against the developed finite-difference-time-domain technique. The interest in circuit design techniques in an IC-compatible fashion in microwave circuits and the introduction of tunable material such as graphene in the THz regime leads to a positive future for the proposed space–time modulation-based crosstalk reduction method.

## Introduction

The concept of Space–time modulated (STM) media has been the subject of intensive research in recent years. Space–time modulation which means varying martial parameters in time and space can break time-reversal symmetry^1^ resulting in frequency conversion, nonreciprocity, and many other exotic effects. Some examples are matching circuits beyond the Bode-Fano limit^2^, non-reciprocal filters^3^, frequency mixers^4^, circulators^5^, and efficient RF front ends^6^. Although there are other methods for implementing microwave devices such as using magnetic materials or biasing a nonlinear element, leveraging STM has specific advantages. Magnetic materials (ferrites in essence) are bulky and incompatible with integrated-circuit technology. On the other hand, a biased element (for example an active transistor) has poor noise performance and power handling concerns^7^. This proves the STM media as one of the candidates for realizing novel and efficient microwave devices.

Although there has been an extensive effort in the design of novel microwave devices using STM media, the literature lacks the application of STM in Electromagnetic Compatibility (EMC) problems. It is for the first time that in this paper the concept of STM has been applied to an EMC issue. The important problem of crosstalk reduction has been addressed in this paper. It is shown that by benefiting the capabilities of STM media, the in-band crosstalk can be reduced efficiently.

The quest for efficient crosstalk reduction techniques is raised by the rapid growth of wireless communication and demand for miniaturized RF front ends, which has led to more compact and denser electronic circuits^8^. As the circuits are designed in smaller footprints and data rates get higher, the issue of unintentional coupling from trace to trace or board to board is becoming increasingly critical^9^.

Many studies have addressed the crosstalk reduction problem. The separation of intruder and victim traces by using via fences or guard traces has been presented in Refs.^10–12^. Although it is an effective technique but hinders the small footprint and integrity of the circuit. In Refs.^13,14^ the crosstalk is reduced by using electromagnetic band gap structures and metamaterials. Both of these methods require defecting the PCB ground plane which in general is not favorable. In another research, authors have benefited from the concept of spoof surface plasmon polaritons to mitigate the crosstalk in differential microstrip lines^15^. It is also interesting to mention some researches that have tackled the problem from a completely different point of view. In Refs.^16,17^ using the concept of linear algebra the crosstalk has been—theoretically—completed by elegant intentional injection of the intruder signal in the victim trace. This method is theoretically interesting but its application to real-world problems is limited.

Different strategies and various research cited here depict the importance of the crosstalk reduction problem, from a scientific perspective and industrial applications. This justifies the necessity for new research and novel effective methods to improve the crosstalk problem.

In this paper, a new paradigm based on the concept of STM media is suggested to tackle the coupling between two adjacent transmission lines. This method leverages the frequency conversion ability of STM transmission lines to reduce the in-band crosstalk. A coupled transmission line, which is a ubiquitous part of an electronic circuit is considered and the crosstalk is mitigated by engineering the space–time modulation of per-unit-length parameters. The working principle relies on the fact that STM can result in a frequency shift in the coupled signal which can be filtered out easily. For any specific coupled transmission line structure, one can reduce the undesired coupled signal lines by elegant designing the space–time modulation parameters as a function of the working frequency and the electrical parameters of the lines. This method is general and can be applied to a wide class of problems. Its verification has been investigated with finite-difference-time-domain (FDTD) as a full-wave method and in a theoretical framework, with good results.

To analyze the problem efficiently, we present a new analytical formulation to predict the STM coupled transmission line behavior fast and accurately. Till now theoretical methods for investigation of space and or time-varying systems can be categorized as follows: some researchers tackle the problem by direct solution of the so-called Telegrapher’s coupled differential equations^18,19^. The Floquet-Bloch approach has been also applied to the problem of wave propagation in STM media^20,21^. However, these methods usually deal with infinitely long transmission lines and the application of arbitrary boundary conditions is usually not an easy task. The new approach presented in this paper is based on the State Space method (SS) which is introduced in the modern control theory of linear systems^22^. The proposed approach expands the voltage and current in terms of spatiotemporal harmonics, which in conjunction with the telegrapher’s equations describe the dynamics of the structure in state space formulation. For the application of this paper, the analysis is restricted to three main harmonics which have been shown to be a good assumption previously in^18,23^ and is known as Coupled Mode Theory (CMT). Using the state space method while benefiting from the coupled mode theory the proposed method is abbreviated as ‘SS-CMT’ throughout this paper. The proposed method is straightforward, easy to formulate, and can be extended to more complex coupled line structures consisting of an arbitrary number of transmission lines with the most general form of the boundary condition. There is also no need for an explicit solution of the wave equation and extraction of the phase constant. The proposed method benefits from the matrix representation which permits exploiting powerful tools developed for matrix processing in software such as MATLAB and Mathematica. The developed analytical method is validated with FDTD which shows very good agreement while being more than 100 times faster.

This paper is organized as follows. “Results” presents the problem geometry and the theoretical framework developed for analyzing the STM transmission lines. Also, some discussion is given on advances and practical realization of STM media. Next in “Verification of the proposed analysis method using FDTD for STM media”, to validate the proposed analytical method, a full-wave numerical technique based on FDTD is developed for simulating coupled STM transmission lines. “Example: a STM coupled transmission line” formulates near and far-end crosstalk in the STM transmission line and introduces the theory for crosstalk reduction besides the physical justification for the proposed method. In “Crosstalk reduction” the applicability of the proposed method for crosstalk reduction is studied by a comprehensive numerical example and its implications are investigated. Finally, “Discussion” concludes the paper.

## Results

### Problem formulation and theoretical analysis for STM structures

This section is devoted to the development of an analytical method for solving the STM coupled transmission line. The main objective of this research is to tackle the crosstalk reduction problem with the aid of space–time modulation. For efficient crosstalk minimization, the modulation parameters should be designed. This gives rise to the need for an efficient, fast and accurate analysis method that can be used in the design process and optimization loop for obtaining the optimal modulation parameters. First, in part A, the geometry of the problem under consideration is presented. Next in part B, the detailed mathematical derivation of the proposed analytical method which is based on the State Space formulation and is supported by Coupled Mode Theory (or SS-CMT) is discussed.

### Problem definition

In this part, the details for analysis of a space–time modulated coupled transmission line is presented. Consider two lines of length d with voltages defined with respect to a common reference conductor. Terminal loads are ${Z}_{S,1(2)}$ and ${Z}_{L,1(2)}$ for line 1(2). Line 1 is excited with a voltage source with temporal frequency ${\omega }_{s}$ and amplitude ${V}_{s}$. The per-unit-length capacitance and inductance matrixes are $[C]$ and $[L]$, where the mutual capacitance ${C}_{m}$ and mutual inductance ${L}_{m}$ are assumed to be space–time modulated as in (1a–1c).

1a $$\left[C\right]=\left[\begin{array}{cc}{C}_{11}+{C}_{m}& {-C}_{m}\\ -{C}_{m}& {C}_{22}+{C}_{m}\end{array}\right], \left[L\right]=\left[\begin{array}{cc}{L}_{11}& {L}_{m}\\ {L}_{m}& {L}_{22}\end{array}\right]$$

1b $${C}_{11}={C}_{110}, {L}_{11}={L}_{110}, {C}_{22}={C}_{220}, {L}_{22}={L}_{220}$$

1c $${C}_{m}={C}_{m0}\left[1+{m}_{C}\mathrm{cos}\left({\omega }_{m}t-{\beta }_{m}z\right)\right]$$

1d $${L}_{m}={L}_{m0}\left[1+{m}_{L}\mathrm{cos}\left({\omega }_{m}t-{\beta }_{m}z+{\phi }_{{m}_{L}}\right)\right]$$

**Figure 1:**
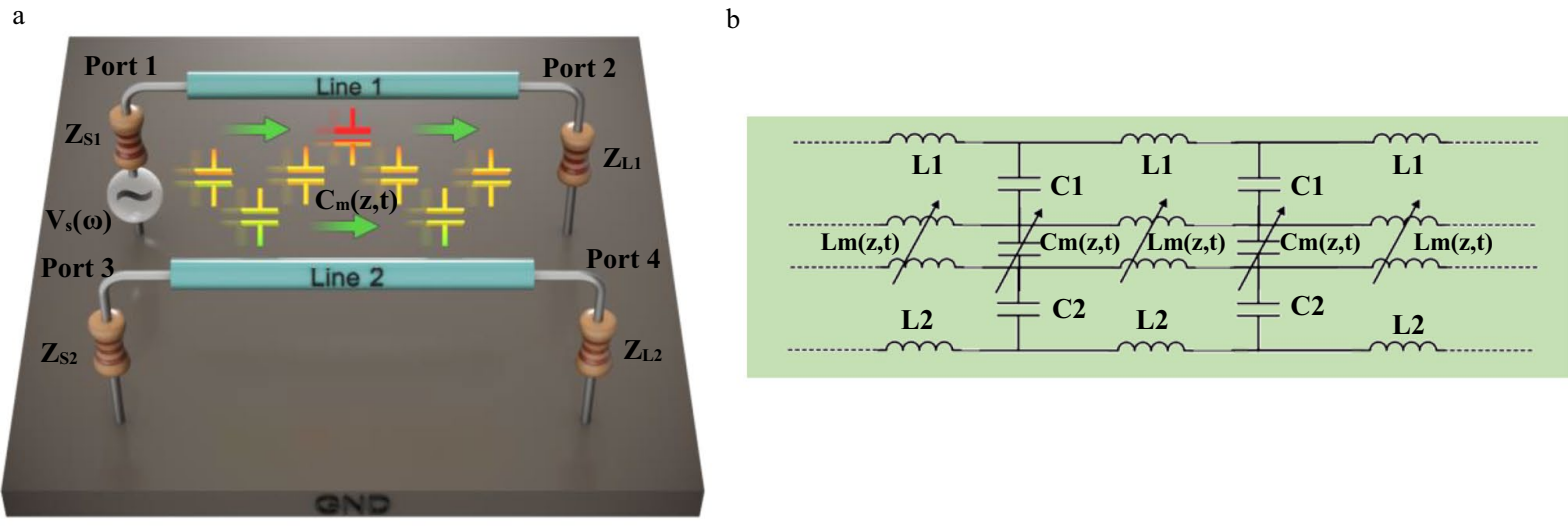
**(a**) Conceptual representation of coupled transmission line with space–time modulated mutual capacitance and mutual inductance. For the sake of clarity the modulated mutual inductance is not shown here. (**b**) Circuit model for a coupled transmission line with space–time modulated mutual capacitance and inductance.

Here ${C}_{11(22)}$ and ${L}_{11(22)}$ are the self-capacitance and self-inductance for line 1(2), respectively. Also, ${C}_{m0}$ and ${L}_{m0}$ are mutual capacitance and mutual inductance when modulation is off. Other parameters are ${\omega }_{m}$ and ${\beta }_{m}$ which represent the temporal and spatial frequency of the modulating wave. Modulation depth for capacitance and inductance are represented with ${m}_{c}$ and ${m}_{L}$, respectively. The generic representation of the STM coupled transmission line under consideration is depicted in Fig. 1a. The governing equations for the structure under consideration are the Telegrapher’s relations in their most general form as in (2a, 2b):

2a $$\frac{\partial \overrightarrow{V}}{\partial z}=-\frac{\partial }{\partial t}\left\{\left[L\right]\overrightarrow{I}\right\}$$

2b $$\frac{\partial \overrightarrow{I}}{\partial z}=-\frac{\partial }{\partial t}\left\{\left[C\right]\overrightarrow{V}\right\}$$

where $\overrightarrow{V}={\left[{V}_{1} {V}_{2}\right]}^{T}$ and $\overrightarrow{I}={\left[{I}_{1} {I}_{2}\right]}^{T}$ are voltage and current vectors respectively. It is well-established that for a transmission line with time-varying capacitance or inductance, a set of new harmonics arise in the line^18^, and their calculation is the subject of this study.

Some statements on the realization of spatiotemporally modulated media should be pointed out. The practical realization of space–time modulated transmission line is an interesting and active research topic^24^. Studies like^4,25^ have addressed the implementation of modulated per-unit-length capacitance. Implementation of per-unit-length modulated inductance is presented in Ref.^18^. Microwave or optical realization of the abovementioned circuits can be accomplished using p-n junctions or varactor-based variable capacitors^25,26^ and varactor-based or thyristor-based variable inductors^27–29^. Loading a transmission line with an array of cascaded omega-metamaterial structures which is tuned by multiple varactors would yield spatiotemporal capacitance and inductance modulation^30^. Similarly, the implementation of a coupled transmission line with modulated mutual capacitance and inductance as in Fig. 1b has been studied. In Ref.^18^ a realization of such a structure has been reported. These studies pave the way for practical applications of spatiotemporal media.

### Mathematical formulation for SS-CMT

In this part, the SS-CMT method which is based on state space formulation and coupled mode theory is presented in detail. Coupled mode theory with the aid of perturbation methods, states that in a small modulation index regime (i.e. m_C_ <  < 1 and m_L_ <  < 1), for solving the problem with an acceptable accuracy one can limit the discussion to three major tones: ω_0_ = ω_s,_ ω_1_ = ω_s_ + ω_m_ and ω_-1_ = ω_s_ − ω_m_^31^. However, we will see that the three-tone assumption gives acceptable results even for bigger modulation indexes. It is important to note that although we have restricted the formulation to structures with two conductors, the SS-CMT has no limitation for the number of conductors and can be used in more general cases. For the intent of this research which is crosstalk reduction and that the case of the two-conductor transmission line is more important, we have restricted the formulation to two-conductor structures. Following the aforementioned assumption, we expand the voltage and current of line “i" as follows:

3a $${V}_{i}\left(z,t\right)=\sum_{u=-1}^{1}\left\{{P}_{u}^{i}\left(z\right){e}^{-j\left({\omega }_{u}t-{\beta }_{u}z\right)}+{P}_{u}^{i*}\left(z\right){e}^{+j\left({\omega }_{u}t-{\beta }_{u}z\right)}\right\}$$

3b $${I}_{i}\left(z,t\right)=\sum_{u=-1}^{1}\left\{{R}_{u}^{i}\left(z\right){e}^{-j\left({\omega }_{u}t-{\beta }_{u}z\right)}+{R}_{u}^{i*}\left(z\right){e}^{+j\left({\omega }_{u}t-{\beta }_{u}z\right)}\right\}.$$

where, u = − 1, 0, 1 and ${\beta }_{u}$ is the spatial frequency at ${\omega }_{u}$. Also ${P}_{u}^{i}(z)$ and ${R}_{u}^{i}(z)$ represent the voltage and current envelope in line “i", i = 1, 2. It is interesting to see that ${\beta }_{0}$, ${\beta }_{+1}$ and ${\beta }_{-1}$ are interrelated. Considering a non-dispersive transmission line over the frequency band of interest, we have:

4 $$\frac{{\omega }_{s}}{{\beta }_{s}}=\frac{{\omega }_{m}}{{\beta }_{m}}=\frac{{\omega }_{1}}{{\beta }_{1}}=\frac{{\omega }_{-1}}{{\beta }_{-1}}$$

which with some mathematical manipulations results in:

5 $${\beta }_{u}= {\beta }_{s}+u{\beta }_{m}$$

In these equations, ${\beta }_{s}$ is the propagation constant of the signal in the absence of modulation and is calculated from the electromagneticarameters of the structure. Using (1a–1d) and (3a, 3b), we can rewrite (2a, 2b) as follows:

6a $$ \begin{gathered} \mathop \sum \limits_{u = - 1}^{1} \left\{ {\left( {\frac{d}{dz}P_{u}^{i} - j\beta_{u} P_{u}^{i} } \right)e^{{ - j\left( {\omega_{u} t - \beta_{u} } \right)}} + \left( {\frac{d}{dz}P_{u}^{i*} + j\beta_{u} P_{u}^{i*} } \right)e^{{ + j\left( {\omega_{u} t - \beta_{u} } \right)}} } \right\} \hfill \\ = - L_{ii} \mathop \sum \limits_{u = - 1}^{1} \left\{ {R_{u}^{i} j\omega_{u} e^{{ - j\left( {\omega_{u} t - \beta_{u} } \right)}} - R_{u}^{i*} j\omega_{u} e^{{ + j\left( {\omega_{u} t - \beta_{u} } \right)}} } \right\} \hfill \\ - L_{m0} \frac{{m_{L} }}{2} e^{{ + j\phi_{mL} }} \mathop \sum \limits_{u = - 1}^{1} \left\{ {R_{u}^{i^{\prime}} j\omega_{u + 1} e^{{ - j\left( {\omega_{u + 1} t - \beta_{u + 1} } \right)}} - R_{u}^{i^{\prime}*} j\omega_{{\left( {u + 1} \right)}} e^{{ + j\left( {\omega_{u + 1} t - \beta_{u + 1} } \right)}} } \right\} \hfill \\ - L_{m0} \frac{{m_{L} }}{2}{ }e^{{ - j\phi_{mL} }} \mathop \sum \limits_{u = - 1}^{1} \left\{ {R_{u}^{{i{^{\prime}}}} j\omega_{u - 1} e^{{ - j\left( {\omega_{u - 1} t - \beta_{u - 1} } \right)}} - R_{u}^{{i{^{\prime}*}}} j\omega_{{\left( {u - 1} \right)}} e^{{ + j\left( {\omega_{u - 1} t - \beta_{u - 1} } \right)}} } \right\} \hfill \\ \end{gathered} $$

6b $$ \begin{gathered} \mathop \sum \limits_{u = - 1}^{1} \left\{ {\left( {\frac{d}{dz}R_{u}^{i} - j\beta_{u} R_{u}^{i} } \right)e^{{ - j\left( {\omega_{u} t - \beta_{u} } \right)}} + \left( {\frac{d}{dz}R_{u}^{i*} + j\beta_{u} R_{u}^{i*} } \right)e^{{ + j\left( {\omega_{u} t - \beta_{u} } \right)}} } \right\} \hfill \\ = - C_{ii} \mathop \sum \limits_{u = - 1}^{1} \left\{ {P_{u}^{i} j\omega_{u} e^{{ - j\left( {\omega_{u} t - \beta_{u} } \right)}} - P_{u}^{i*} j\omega_{u} e^{{ + j\left( {\omega_{u} t - \beta_{u} } \right)}} } \right\} \hfill \\ - C_{m0} \frac{{m_{C} }}{2} \mathop \sum \limits_{u = - 1}^{1} \left\{ {\left( {P_{u}^{i^{\prime}} - P_{u}^{i} } \right)j\omega_{u + 1} e^{{ - j\left( {\omega_{u + 1} t - \beta_{u + 1} } \right)}} - \left( {P_{u}^{{i^{\prime}*}} - P_{u}^{i*} } \right)j\omega_{{\left( {u + 1} \right)}} e^{{ + j\left( {\omega_{u + 1} t - \beta_{u + 1} } \right)}} } \right\} \hfill \\ - C_{m0} \frac{{m_{C} }}{2}{ }\mathop \sum \limits_{u = - 1}^{1} \left\{ {\left( {P_{u}^{{i{^{\prime}}}} - P_{u}^{i} } \right)j\omega_{u - 1} e^{{ - j\left( {\omega_{u - 1} t - \beta_{u - 1} } \right)}} - \left( {P_{u}^{{i^{\prime}{*}}} - P_{u}^{{i{*}}} } \right)j\omega_{{\left( {u - 1} \right)}} e^{{ + j\left( {\omega_{u - 1} t - \beta_{u - 1} } \right)}} } \right\} \hfill \\ \end{gathered} $$

where ($i=1, {i}^{^{\prime}}=2)$ or ($i=2, {i}^{^{\prime}}=1)$. Taking advantage the orthogonality property of exponential functions and using (6a) and (6b), yields:

7a $$\frac{d}{dz}{P}_{u}^{i}=j{\beta }_{u}{P}_{u}^{i}-{L}_{ii}{R}_{u}^{i}j{\omega }_{u}-{L}_{m0}\frac{{m}_{L}}{2} {e}^{+j{\phi }_{mL}} j{\omega }_{u}{R}_{u+1}^{i\mathrm{^{\prime}}}-{L}_{m0}\frac{{m}_{L}}{2} {e}^{-j{\phi }_{mL}} j{\omega }_{u}{R}_{u-1}^{i\mathrm{^{\prime}}}$$

7b $$\frac{d}{dz}{R}_{u}^{i}=j{\beta }_{u}{R}_{u}^{i}-{C}_{ii}{P}_{u}^{i}j{\omega }_{u}-{C}_{m0}\frac{{m}_{C}}{2}j{\omega }_{u}\left({P}_{u+1}^{i\mathrm{^{\prime}}}-{P}_{u+1}^{i}\right)-{C}_{m0}\frac{{m}_{C}}{2}j{\omega }_{u}\left({P}_{u-1}^{i\mathrm{^{\prime}}}-{P}_{u-1}^{i}\right)$$

Here we just keep harmonics with order 0, + 1 or − 1 and drop the higher order terms arising in multiplication process. By inspecting (7a, 7b) one can put the equations in the well-known form of state space formulation as in (8a–8c)^22^:

8a $$\frac{d}{dz}\overrightarrow{X}=[A]\overrightarrow{X}$$

8b $$\overrightarrow{X}=\left[{P}_{-1}^{1}, {P}_{0}^{1}, {P}_{1}^{1},{P}_{-1}^{2}, {P}_{0}^{2}, {P}_{1}^{2},{R}_{-1}^{1}, {R}_{0}^{1}, {R}_{1}^{1},{R}_{-1}^{2}, {R}_{0}^{2}, {R}_{1}^{2}\right]$$

8c $$\left[A\right]=-j\left[\begin{array}{cccc}{\beta }_{-1}& 0& \dots & 0\\ 0& {\beta }_{0}& {-L}_{m0}\frac{{m}_{L}}{2} {e}^{+j{\phi }_{mL}}{\omega }_{0}& -{L}_{m0}\frac{{m}_{L}}{2} {e}^{+j{\phi }_{mL}} {\omega }_{0}\\ \vdots & 0& \ddots & 0\\ 0& {C}_{m0}\frac{{m}_{C}}{2}{\omega }_{u}& \dots & {\beta }_{1}\end{array}\right]$$

The complete form of the matrix [A] is presented in Supplementary S1. In (8a–8c), $\overrightarrow{X}$ is the state vector, containing the sought-for voltage and current envelopes over the line while [A] bears the coupled line circuital parameters used in the description of the dynamic of the system. State space is a powerful and well-established strategy in control theory for linear systems. Various analytically robust and numerically fast and accurate methods have been developed for solving equations described in state space formalism^32^. Once we formulated our electromagnetic problem in the proper form of state space formulation, we can exploit these well-developed methods to analyze the problem effectively.

To have a complete solution to the problem and incorporate the terminal effects (boundary conditions), we have to compute the following:

9 $$\overrightarrow{X}\left(d\right)=\mathrm{exp}\left(-\left[A\right]d\right)\overrightarrow{X}(0)$$

Several methods have addressed the computation of exponential of a matrix which arises in (9) such as Laplace transform, expansion in power series, and Cayley–Hamilton theorem^33^, which can be used efficiently. The boundary conditions of the problem are:

10a $$\overrightarrow{V}\left(d\right)=\left[{Z}_{L}\right]\overrightarrow{I}(d)$$

10b $${\overrightarrow{V}}_{s}-\overrightarrow{V}\left(0\right)=\left[{Z}_{S}\right]\overrightarrow{I}(0)$$

where $\left[{Z}_{S}\right]$ and $\left[{Z}_{L}\right]$ are the source and load matrix respectively. Simultaneous solution of (9) and (10a, 10b), yields the state vector in the terminals of the structure, i.e. $\overrightarrow{X}(0)$ and $\overrightarrow{X}(d)$. Afterward voltage and currents can be computed in any arbitrary position like “z” in lines as calculated in (11). It should be noted that the voltage and current vectors $\overrightarrow{V}$ and $\overrightarrow{I}$ in (10a, 10b) are related to the sought-for envelopes P and R by (3). The state vector X compactly denotes the voltage and current envelopes as in (8a–8c).

11 $$\overrightarrow{X}\left(z\right)=\mathrm{exp}\left(-\left[A\right]z\right)\overrightarrow{X}(0)$$

### Verification of the proposed analysis method using FDTD for STM media

To validate the developed theoretical analysis method (SS-CMT), a full-wave simulation technique is employed. Because of the time-variant nature of the problem, FDTD is chosen which has been proven to be useful in handling electromagnetic complex structures^34^. As the structure under study is of time-variant nature, the conventional frequency domain techniques such as FEM, MoM, etc., cannot be used. Here, we develop an FDTD formulation to handle the STM coupled transmission line. The governing relations are the telegrapher’s equations for which the time dependency should be taken into account as is explicitly stated in (2a, 2b). For ease of notation we have used the nomenclature in (12) for the [L] and [C] to derive FDTD equations:

12 $$\left[C\right]=\left[\begin{array}{cc}{C}_{11}& {-C}_{12}\\ {-C}_{21}& {C}_{22}\end{array}\right], \left[L\right]=\left[\begin{array}{cc}{L}_{11}& {L}_{12}\\ {L}_{21}& {L}_{22}\end{array}\right]$$

**Figure 2:**
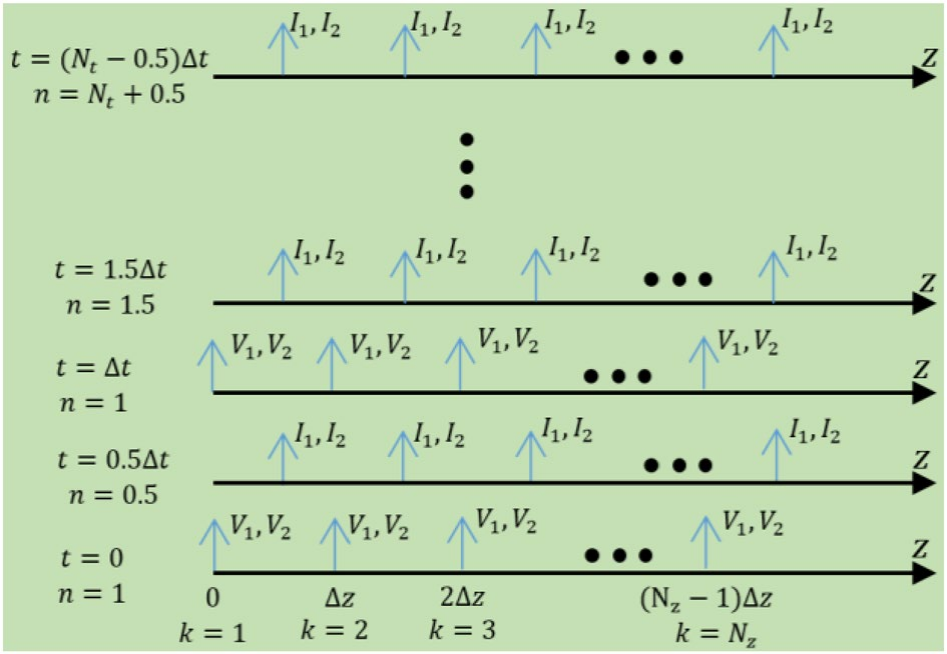
Representation of FDTD scheme for full wave simulation of space–time coupled transmission lines. V_1(2)_ and I_1(2)_ represent the voltage and current in line 1(2), respectively.

After discretizing the structure along the line into N_z_ spatial meshes and N_t_ temporal sample, some mathematical manipulation results in the system of update difference equations. The general representation of the FDTD cell for the coupled line is shown in Fig. 2. Equations (13a, 13b) and (14a, 14b) are for currents and voltages update equations, respectively.

13a $${I}_{1}^{n+0.5}\left(k\right)\left[\frac{{p}_{1}}{\Delta t}+0.5{p}_{2}\right]+{I}_{2}^{n+0.5}\left(k\right)\left[0.5{p}_{3}\right]=-{I}_{1}^{n-0.5}\left(k\right)\left[0.5{p}_{2}-\frac{{p}_{1}}{\Delta t }\right]-{I}_{2}^{n-0.5}\left(k\right)\left[0.5{p}_{3}\right]+{p}_{0}$$

13b $${I}_{1}^{n+0.5}\left(k\right)\left[0.5{q}_{2}\right]+{I}_{2}^{n+0.5}\left(k\right)\left[\frac{{q}_{1}}{\Delta t}+0.5{q}_{3}\right]=-{I}_{1}^{n-0.5}\left(k\right)\left[0.5{q}_{2}\right]-{I}_{2}^{n-0.5}\left(k\right)\left[0.5{p}_{3}-\frac{{q}_{1}}{\Delta t }\right]+{q}_{0}$$

14a $${V}_{1}^{n+0.5}\left(k\right)\left[\frac{{x}_{1}}{\Delta t}+0.5{x}_{2}\right]+{V}_{2}^{n+0.5}\left(k\right)\left[0.5{x}_{3}\right]=-{V}_{1}^{n-0.5}\left(k\right)\left[0.5{x}_{2}-\frac{{x}_{1}}{\Delta t }\right]-{V}_{2}^{n-0.5}\left(k\right)\left[0.5{x}_{3}\right]+{x}_{0}$$

14b $${V}_{1}^{n+0.5}\left(k\right)\left[0.5{y}_{2}\right]+{V}_{2}^{n+0.5}\left(k\right)\left[\frac{{y}_{1}}{\Delta t}+0.5{y}_{3}\right]=-{V}_{1}^{n-0.5}\left(k\right)\left[0.5{y}_{2}\right]-{V}_{2}^{n-0.5}\left(k\right)\left[0.5{y}_{3}-\frac{{y}_{1}}{\Delta t }\right]+{y}_{0}$$

Here, the superscript n and the argument k represent the time step and position, respectively. Coefficient terms ${p}_{i}, {q}_{i}, {x}_{i,}$ and ${y}_{i}$ where i = 0, 1, 2 bear the structure electromagnetic parameters and are presented in Supplementary S1. After developing the FDTD it is used to validate the proposed analytical method. An examples have been worked out for this purpose. Good agreement shows the consistency of the proposed analytical method which is accurate while much faster than FDTD.

### Example: a STM coupled transmission line

The geometry of the problem is similar to the structure in Fig. 1. The source V_s_ excites the structure with frequency ω_s_ = 2π × 1.5 GHz. The length of the lines is considered d = 2λ_0_ and the capacitance and inductance matrixes are as follows: C_11_ = C_22_ = 116.95 pF/m, C_12_ = C_21_ = 46.6 pF/m, L_11_ = L_22_ = 248.45 nH/m and L_12_ = L_21_ = 100 nH/m.

**Figure 3:**
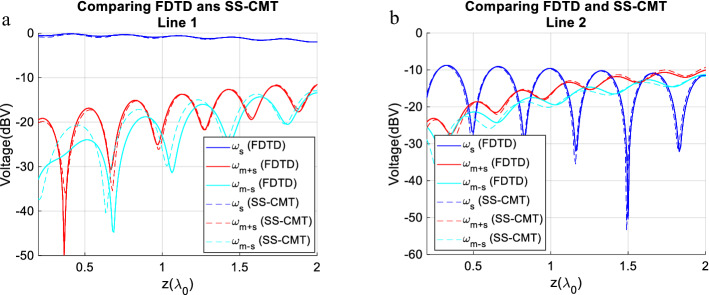
The evolution of the main three harmonics along the lines, (**a**) line 1 and (**b**) line 2. The modulation give rise to harmonics.

The mutual capacitance and inductance are modulated in space and time as follows. Referring to (1c) and (1d) the modulation parameters are: m_C_ = 0.35, m_L_ = 0.35, Φ_mL_ = 0 and ω_m_ = 2π × 200 MHz. It is noteworthy to say that the proposed method is general and the choice of ω_m_ = 2π × 200 MHz is arbitrary. We have tested different scenarios and other frequencies can be selected. For the sake of brevity, one typical example is addressed here aimed to show the applicability of the SS-CMT. In Fig. 3 the magnitude of the three main harmonics i.e., ω_s_ + ω_m_, ω_s_ − ω_m_, and ω_s_ are presented. The way that harmonics evolve along the line is very insightful. As the wave propagates, the energy couples from the main harmonic to adjacent ones. Good consistency between the results of FDTD and that of SS-CMT is clear in Fig. 3 which demonstrates an acceptable level of accuracy. On the other hand, SS-CMT is much faster than FDTD. In this example, the runtime for FDTD is about 460 s while SS-CMT just took one second to give the results. This makes the SS-CMT a suitable tool for design problems that require multiple-time analysis of the forward problem. The computer used for this example has 32 GB RAM and is equipped with a 3.2 GHz core i7 Intel CPU. It is speculated that some discrepancies observed in Fig. 3 is due to the residual error in computation of Fast Fourier Transform (FFT) of FDTD results.

### Numerical example

In this section, the various implications of the proposed method for energy coupling control are discussed through a detailed example. The beginning is dedicated to the introduction of the optimization setup which calculates the effective modulation parameters. In the remainder the crosstalk reduction has been tackled. And at the end, a parametric study on modulation parameters is presented. Effect of various parameters on the line response are investigated and some conclusions are drawn for further designs.

For the remainder of this section, consider a coupled transmission line with a length d = 20 cm, for which the per-unit-length capacitance and inductance matrixes is given in (15).

15 $$\left[C\right]=\left[\begin{array}{cc}493.3& -60.3\\ -60.3& 493.3\end{array}\right]\frac{pF}{m}, [L]=\left[\begin{array}{cc}204& 49.7\\ 49.7& 204\end{array}\right]\frac{nH}{m}$$

**Figure 4:**
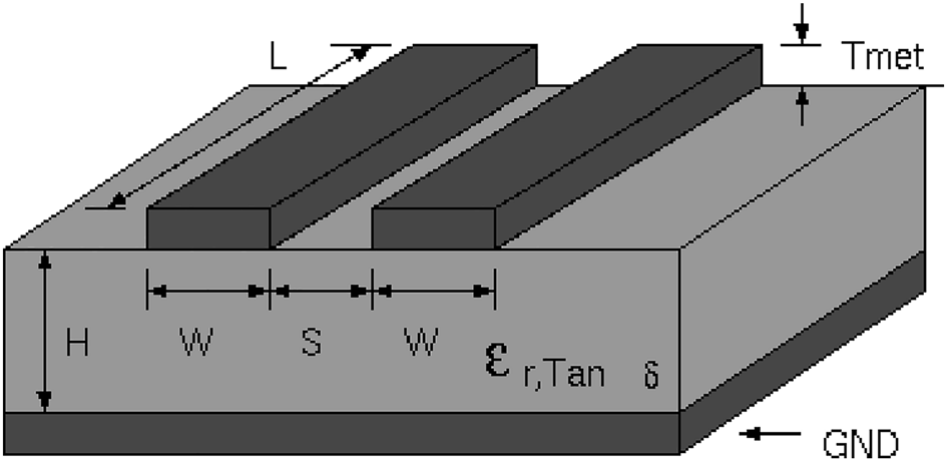
A microstrip as an example of physical realization of a coupled transmission line, with parameters W = 1.2 mm, S = 0.1 mm, H = 0.35 mm and substrate dielectric constant 12.

This line has a physical realization as a microstrip line with the following parameters: W = 1.2 mm, S = 0.1 mm, H = 0.35 mm and substrate dielectric constant 12 as shown in Fig. 4^36^. The main line (line 1) is excited with frequency ω_s_ = 2π × 1.5 rad.GHz with a voltage source of unite amplitude and the ports are terminated to 50 Ω loads.

We are interested in the computation of the coupled energy (or crosstalk) in the victim (line 2) and minimizing it through appropriate spatiotemporally modulating the mutual capacitance and inductance. The mutual capacitance and inductance are space–time modulated as in (1c) and (1d).

Using^35^, the formal definition of far-end and near-end crosstalk in a coupled transmission line is defined in (16a, 16b). Consider a coupled transmission line as in Fig. 1.

16a $$\mathrm{Far}-\mathrm{end}=20\mathrm{log}\left(\frac{{V}_{4}}{{V}_{s}}\right)$$

16b $$\mathrm{Near}-\mathrm{end}=20\mathrm{log}\left(\frac{{V}_{3}}{{V}_{s}}\right)$$

Here V_4_, V_3_, and V_s_ are the voltages of port 4, port 3, and the source. By inspecting (11) one can see that V_4_ and V_3_ are function of the matrix [A]. The matrix [A] which its complete form is presented in Supplementary S1, has an explicit dependency on the modulation parameters ${m}_{L}, {m}_{C}, {\omega }_{m}$ and ${\phi }_{{m}_{L}}$. This clearly shows one can have control over V_4_ and V_3_ by spatiotemporally modulating the structure which in fact enables engineering the far-end or near-end crosstalk.

### The optimization setup

For finding the modulation parameters for an efficient crosstalk reduction an optimization process is adopted. Although Eqs. (10a, 10b) and (11) support the theoretical background for the calculation of the crosstalk, there is no closed form solution for the selection of modulation parameters. In fact the problem at hand is a “design” problem, where we have to select the design parameters (here the modulation indexes and frequency) in order to get a desired result (the reduced crosstalk). The optimizer engine calculates the crosstalk for a variety set of modulation parameters and finds the best answer to fit the problem requirements. In this process, the forward problem has to be solved numerous times. Using the proposed analytical formulation (SS-CMT) which is fast and accurate makes the optimization process feasible. The optimizer maximizes the difference between $\left|{V}_{3}^{mod off}\right|$ and $\left|{V}_{3}^{mod on}\right|$ for near-end crosstalk reduction or $\left|{V}_{4}^{mod off}\right|$ and $\left|{V}_{4}^{mod on}\right|$ for far-end crosstalk reduction. Also in order not to perturb the main line signal integrity, a constraint has to be defined for port 2 as: $\left|{V}_{2}^{mod on}\right| \cong \left| {V}_{2}^{mod off}\right|$. Here the subscript denotes the number of port. And superscript denotes whether modulation is on or off. An appropriate goal function is suggested in (17).

17 $$ \begin{gathered} Cost = \left( {\left| {V_{3\left( 4 \right)}^{mod on} } \right| - \left| {V_{3\left( 4 \right)}^{mod off} } \right|} \right)^{ - 2} \hfill \\ Costraint = \left( {\left| {{ }V_{2}^{mod on} } \right| - \left| {{ }V_{2}^{mod off} } \right|} \right) < \varepsilon \hfill \\ \end{gathered} $$

where, $\varepsilon$ is the maximum tolerable signal deterioration in the main line due to the application of crosstalk reduction.

### Crosstalk reduction

The schematic presentation of the problem is presented in Fig. 1, where the goal is to reduce the coupled energy in port 3 or port 4. Here as an example port 3 is considered. In order to reduce the crosstalk, we have to minimize the cost function defined in (17) for $\left|{V}_{3}^{mod off}\right|$ and $\left|{V}_{3}^{mod on}\right|$. Here the well-known Particle Swarm Optimization (PSO) algorithm which is a popular method in the microwave design community^37^ was used. The simulation was performed on an Intel(R) Core(TM) i5, 3230 M, CPU running at 2.60 GHz and 12 GB of RAM which lasts only 60 s to converge. The modulation parameters in (18) give rise to good crosstalk mitigation:

18 $${m}_{C}=0.2,{m}_{L}=0.7, {\omega }_{m}=2\pi .122 MHz,{\phi }_{mL}=\frac{3\pi }{5}$$

**Figure 5:**
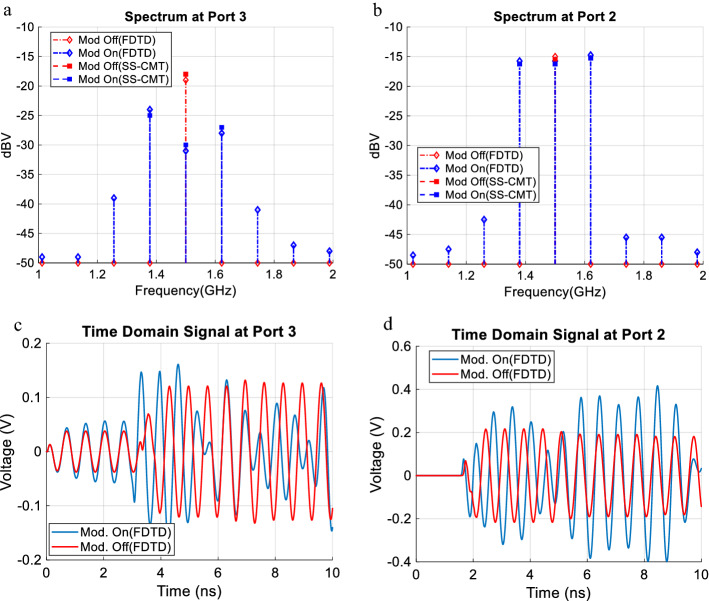
Comparing response of the modulated and unmodulated structure: (**a**) frequency spectrum for port 3 (the near-end crosstalk in line 2), (**b**) frequency spectrum for port 2 (the main signal in line 1), (**c**) time domain signal at port 3 and (**d**) Time domain signal at port 2. Some 12 dB crosstalk reduction is observed.

Comparing the frequency spectrum in port 3 for modulated and unmodulated cases, a 12 dB crosstalk reduction is observed, Fig. 5a.

It is well established that the frequency conversion effect occurs when the main wave and modulating wave have close phase velocities and hence are nearly phase matched^24^. For the near-end case the undesired energy is coupled into line 2 and propagates backward to reach port 3. Therefore the phase front of the modulating wave should be of the form $\phi = {\omega }_{m}t+{\beta }_{m}z$ which means a–z directed wave propagation.

**Figure 6:**
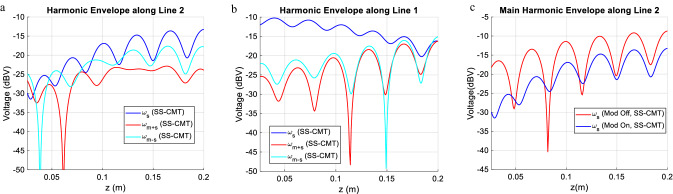
The evolution of the main three harmonics envelopes along the (**a**) line 2 and (**b**) line 1. (**c**) Comparing the envelope of the main harmonic in line 2 for the modulated and unmodulated structure.

Although space–time modulating the line reduces the in-band coupled signal, it has unfavorable adverse effects on the main line signal propagation which has to be controlled to make the method of practical importance. One should take into account these effects while designing the space–time modulation parameters. In Fig. 5b which shows the spectrum of the output of line 1, it is seen that the signal in port 2 is reduced by only 0.5 dB which is not problematic from the signal integrity viewpoint for a wide variety of applications. This means that the proposed method does not have an adverse effect on the main line signal quality. The time domain signals in port 2 and port 3 are presented in Fig. 5c,d for more insight. The introduction of new frequency content for modulated structures is clearly observed from the time domain signal. In order to elaborate more on the details, we present the harmonic evolution on line 1 and line 2 in Fig. 6. It is seen as the signal propagates along the structure more and more energy is converted from the main frequency to other harmonics. It is well known that the amplitude variation in the coupled line takes a standing wave like pattern, having peaks and nulls along the line which is highlighted in Fig. 6b. The interesting observation is that space–time modulating not only affects the amplitude of the main harmonic in line 2 but also alters the position of peaks and nulls. This is a very important effect that can help reduce the crosstalk. This phenomena is emphasized in Fig. 6c which compares the envelope of the main harmonic magnitude in line 2 for the modulated and unmodulated structure. It is seen in the near end the nulls are displaced.

### Parametric study of space time modulation

**Figure 7:**
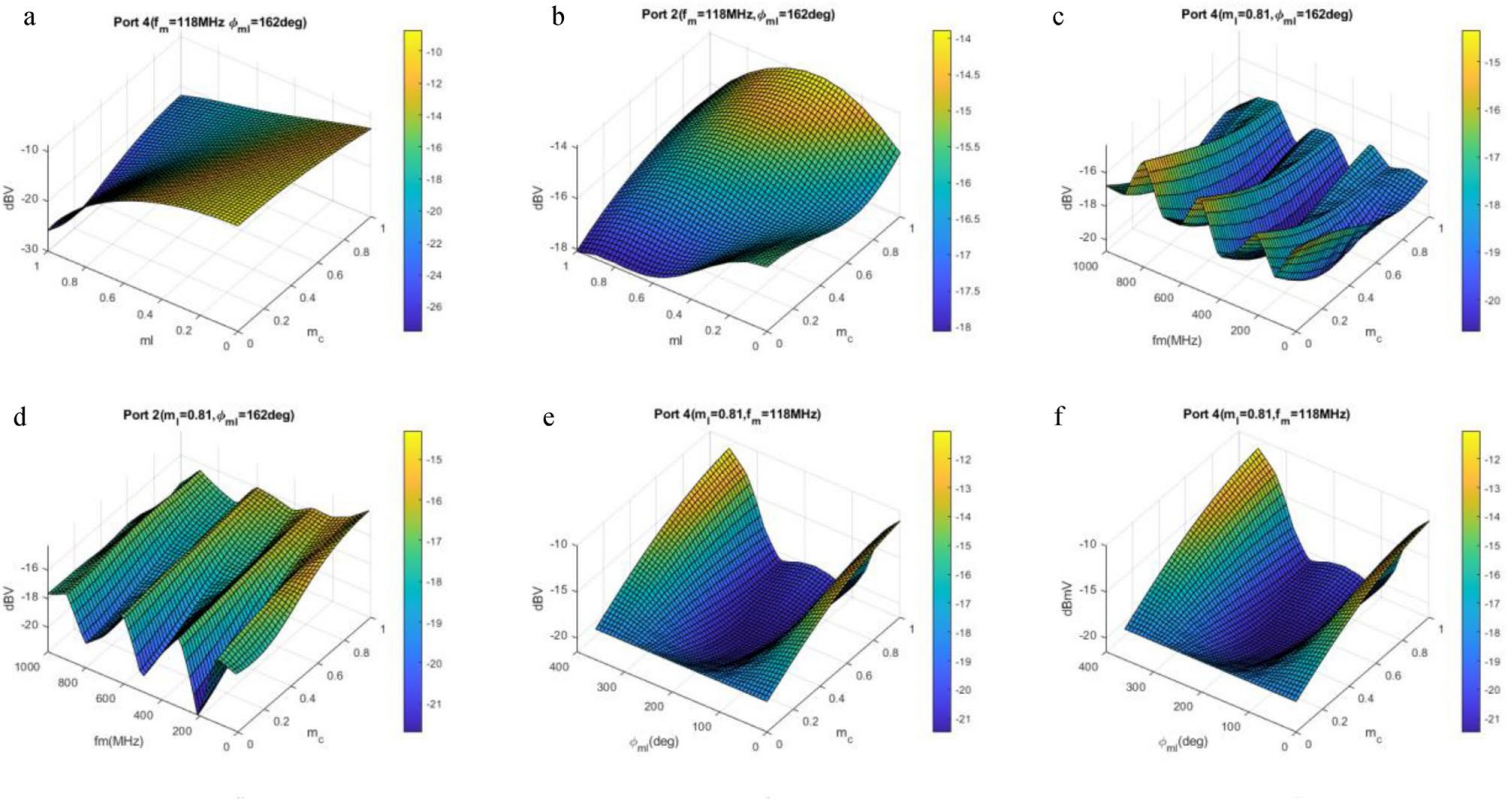
Parametric study of crosstalk versus different parameters, investigating effect of capacitive modulation index and, (**a**) inductive modulation index in port 4, (**b**) inductive modulation index in port 2, (**c**) modulation frequency in port 4, (**d**) modulation frequency in port 2, (**e**) inductive modulation phase in port 4 and (**f**) inductive modulation phase in port 2. Signal in port 4 represents the far-end crosstalk and port 2 the main line signal.

In order to get more insight into the physics of the problem, a thorough parametric study has been conducted. Considering the modulation notation as in (1a–1d), the four parameters under consideration are capacitive modulation index (m_C_), inductive modulation index (m_L_), modulation phase (ϕ_mL_), and modulation frequency (f_m_). Results are presented in Fig. 7 by 3D plots. Each plot represents a sweep of two parameters while the other two are constants. The z-axis shows the amplitude of the main harmonic in the ports in dBV. For the sake of brevity, the main line output (port 2) and the far-end crosstalk (port 4) are considered.

Capacitive modulation m_C_ has been repeated in all the graphs as one of the independent variables. The other independent variable in each graph is m_L_ (a and b), f_m_ (c and d) or ϕ_mL_ (e and f). For each scenario, one graph is dedicated to the output of line 2 (port 4) and the other graph to the output of line 1 (port 2). To elaborate more on the behavior of the structure, we start with modulation indexes. Generally speaking the stronger the modulation index (m_C_ or m_L_) the more energy conversion effect occurs which means higher crosstalk reduction. Observing Fig. 7a confirms this speculation which shows smaller crosstalk for bigger modulation indexes. But the effect of modulation frequency and modulation phase is more interesting and a bit more sophisticated. By inspecting Fig. 7c one can observe an oscillatory behavior of crosstalk versus the modulation frequency. The modulation frequency is swept from 10 to 1000 MHz. For a better crosstalk reduction one should choose the modulation frequency in nulls of the graph while taking into account the main line voltage not to be perturbed unacceptably. On the other hand Fig. 7e shows that the best value for modulation phase is around 180°. In this region the crosstalk is reduced while the main line voltage has the lowest attenuation.

## Discussion

In this paper, we proposed an STM based crosstalk reduction technique in a coupled transmission line. It was shown that by proper spatiotemporally modulating the per-unit-length parameters the coupled energy to the victim line at the frequency of interest can be minimized. This is made possible by wisely taking advantage of the frequency conversion ability of STM media. In order to obtain the optimal modulation scheme for the defined goals a fast and accurate analysis method was developed to tackle the problem. The developed analytical method is based on the state space formulation and coupled mode theory (SS-CMT) which effectively analyzes STM structures. The proposed analytical method was validated by the results of FDTD, as a full wave simulation technique. Speeding up the analysis more than 100 times in conjunction with good accuracy makes the SS-CMT a versatile candidate method for tackling design problems involving STM structures. After giving some physical justification elaborating on the mechanism of the crosstalk reduction technique by STM media, the applicability of the proposed technique was studied through a comprehensive numerical example. This example considers a coupled microstrip line as a physical implementation of coupled transmission line, demonstrating more than 10 dB crosstalk reduction in near-end and far-end terminals. Based on the recent developments in the implementation of the STM media and the feasibility of the proposed method using space–time modulation, a new promising paradigm is set forward for handling electromagnetic compatibility issues like crosstalk reduction.

## Supplementary Information

Supplementary Information.

## Data Availability

All data generated or analysed during this study are included in this published article and its supplementary information files.
