# Supplementary material for: Leveraging space–time modulation for energy coupling control in electromagnetic coupled transmission lines structures
Source: Sci Rep. 2023 Feb 2;13:1930. doi: 10.1038/s41598-023-28925-1 (PMC9895445; doi:10.1038/s41598-023-28925-1)
Supplement: Supplementary file 1 — Supplementary Information. [file 41598_2023_28925_MOESM1_ESM.pdf]

Supplementary File

The complete form of the matrix [A] which encompasses the electromagnetic properties of the structure can be deduced from (7). The matrix [A] is a square matrix which for the sake of demonstration we use a simple decomposition: $[A] = [\,[A_1]_{12x6}\ [A_2]_{12x6}]$. Here $[A_1]_{12x6}$ and $[A_2]_{12x6}$ are given in (S1) and (S2). The parameters used in (S1) and (S2) are defined in (1).

$$[A_1] = j\begin{bmatrix} \beta_{-1} & 0 & 0 & 0 & 0 & 0 \\ 0 & \beta_0 & 0 & 0 & 0 & 0 \\ 0 & 0 & \beta_1 & 0 & 0 & 0 \\ 0 & 0 & 0 & \beta_{-1} & 0 & 0 \\ 0 & 0 & 0 & 0 & \beta_0 & 0 \\ 0 & 0 & 0 & 0 & 0 & \beta_1 \\ -C_{11}\omega_{-1} & \frac{m_C}{2}C_{m0}\omega_{-1} & 0 & 0 & -\frac{m_C}{2}C_{m0}\omega_{-1} & 0 \\ \frac{m_C}{2}C_{m0}\omega_0 & -C_{11}\omega_0 & \frac{m_C}{2}C_{m0}\omega_0 & -\frac{m_C}{2}C_{m0}\omega_0 & 0 & -\frac{m_C}{2}C_{m0}\omega_0 \\ 0 & \frac{m_C}{2}C_{m0}\omega_1 & -C_{11}\omega_1 & -\frac{m_C}{2}C_{m0}\omega_1 & 0 & 0 \\ 0 & -\frac{m_C}{2}C_{m0}\omega_{-1} & 0 & -C_{22}\omega_{-1} & \frac{m_C}{2}C_{m0}\omega_{-1} & 0 \\ -\frac{m_C}{2}C_{m0}\omega_0 & 0 & -\frac{m_C}{2}C_{m0}\omega_0 & \frac{m_C}{2}C_{m0}\omega_0 & -C_{22}\omega_0 & \frac{m_C}{2}C_{m0}\omega_0 \\ 0 & -\frac{m_C}{2}C_{m0}\omega_1 & 0 & 0 & \frac{m_C}{2}C_{m0}\omega_1 & -C_{22}\omega_1 \end{bmatrix} \quad \text{(S1)}$$

$$[A_2] = j\begin{bmatrix} -L_{11}\omega_{-1} & 0 & 0 & 0 & -\frac{m_L}{2}L_{m0}\omega_{-1}e^{j\Phi_{mL}} & 0 \\ 0 & -L_{11}\omega_0 & 0 & -\frac{m_L}{2}L_{m0}\omega_0e^{-j\Phi_{mL}} & 0 & -\frac{m_L}{2}L_{m0}\omega_0e^{j\Phi_{mL}} \\ 0 & 0 & -L_{11}\omega_1 & 0 & -\frac{m_L}{2}L_{m0}\omega_1e^{-j\Phi_{mL}} & 0 \\ 0 & -\frac{m_L}{2}L_{m0}\omega_{-1}e^{j\Phi_{mL}} & 0 & -L_{22}\omega_{-1} & 0 & 0 \\ -\frac{m_L}{2}L_{m0}\omega_0e^{-j\Phi_{mL}} & 0 & -\frac{m_L}{2}L_{m0}\omega_0e^{j\Phi_{mL}} & 0 & -L_{22}\omega_0 & 0 \\ 0 & -\frac{m_L}{2}L_{m0}\omega_1e^{-j\Phi_{mL}} & 0 & 0 & 0 & -L_{22}\omega_1 \\ \beta_{-1} & 0 & 0 & 0 & 0 & 0 \\ 0 & \beta_0 & 0 & 0 & 0 & 0 \\ 0 & 0 & \beta_1 & 0 & 0 & 0 \\ 0 & 0 & 0 & \beta_{-1} & 0 & 0 \\ 0 & 0 & 0 & 0 & \beta_0 & 0 \\ 0 & 0 & 0 & 0 & 0 & \beta_1 \end{bmatrix} \quad \text{(S2)}$$

Update equations for the developed FDTD method are derived by replacing the time and position derivative in Telegrapher's equations with central difference formula.

$$f'(x) = \frac{f(x+\Delta x) - f(x-\Delta x)}{2\Delta x} \quad \text{(S3)}$$

After some mathematical manipulations, the coefficients of FDTD equations in (13) and (14) read as follows:

$$\begin{aligned} p_0 &= \frac{L_{12}^n(k)}{\Delta z}[V_2^n(k+1) - V_2^n(k)] - \frac{L_{22}^n(k)}{\Delta z}[V_1^n(k+1) - V_1^n(k)] \\ p_1 &= L_{11}^n(k)L_{22}^n(k) - L_{21}^n(k)L_{12}^n(k) \\ p_2 &= \dot{L}_{11}^n(k)L_{22}^n(k) - \dot{L}_{21}^n(k)L_{12}^n(k) \\ p_3 &= \dot{L}_{12}^n(k)L_{22}^n(k) - \dot{L}_{22}^n(k)L_{12}^n(k) \end{aligned} \quad \text{(S4)}$$

$$\begin{aligned} q_0 &= \frac{L_{21}^n(k)}{\Delta z}[V_1^n(k+1) - V_1^n(k)] - \frac{L_{11}^n(k)}{\Delta z}[V_2^n(k+1) - V_2^n(k)] \\ q_1 &= L_{11}^n(k)L_{22}^n(k) - L_{21}^n(k)L_{12}^n(k) \\ q_2 &= \dot{L}_{21}^n(k)L_{22}^n(k) - \dot{L}_{11}^n(k)L_{21}^n(k) \\ q_3 &= \dot{L}_{22}^n(k)L_{11}^n(k) - \dot{L}_{12}^n(k)L_{21}^n(k) \end{aligned} \quad \text{(S5)}$$

$$x_0 = \frac{C_{12}^{n-0.5}(k)}{\Delta z}\left[I_2^{n-0.5}(k+1) - I_2^{n-0.5}(k)\right] - \frac{C_{22}^{n-0.5}(k)}{\Delta z}\left[I_1^{n-0.5}(k+1) - I_1^{n-0.5}(k)\right] \quad \text{(S6)}$$

$$
\begin{aligned}
x_1 &= C_{11}^{n-0.5}(k)C_{22}^{n-0.5}(k) - C_{21}^{n-0.5}(k)C_{12}^{n-0.5}(k)\\
x_2 &= \dot{C}_{11}^{n-0.5}(k)C_{22}^{n-0.5}(k) - \dot{C}_{21}^{n-0.5}(k)C_{12}^{n-0.5}(k)\\
x_3 &= \dot{C}_{12}^{n-0.5}(k)C_{22}^{n-0.5}(k) - \dot{C}_{22}^{n-0.5}(k)C_{12}^{n-0.5}(k)
\end{aligned}
$$

$$
\begin{aligned}
y_0 &= \frac{C_{21}^{n-0.5}(k)}{\Delta z}\left[I_1^{n-0.5}(k+1) - I_1^{n-0.5}(k)\right] - \frac{C_{11}^{n-0.5}(k)}{\Delta z}\left[I_2^{n-0.5}(k+1) - I_2^{n-0.5}(k)\right]\\
y_1 &= C_{11}^{n-0.5}(k)L_{22}^{n-0.5}(k) - C_{21}^{n-0.5}(k)L_{12}^{n-0.5}(k)\\
y_2 &= \dot{C}_{21}^{n-0.5}(k)C_{22}^{n-0.5}(k) - \dot{C}_{11}^{n-0.5}(k)C_{21}^{n-0.5}(k)\\
y_3 &= \dot{C}_{22}^{n-0.5}(k)C_{11}^{n-0.5}(k) - \dot{C}_{12}^{n-0.5}(k)C_{21}^{n-0.5}(k)
\end{aligned}
\tag{S7}
$$

Here coefficient terms p_i, q_i, x_i and y_i where i = 0, 1 and 2 bear the structure electromagnetic parameter and are used in (13) and (14) for FDTD update equations. Also the $(\dot{\ })=\frac{\partial}{\partial t}$ denote the time derivative operator.
